# Development and validation of a prognostic multivariable model to predict insufficient clinical response to methotrexate in rheumatoid arthritis

**DOI:** 10.1371/journal.pone.0208534

**Published:** 2018-12-10

**Authors:** Maurits C. F. J. de Rotte, Saskia M. F. Pluijm, Pascal H. P. de Jong, Maja Bulatović Ćalasan, Nico M. Wulffraat, Angelique E. A. M. Weel, Jan Lindemans, J. M. W. Hazes, Robert de Jonge

**Affiliations:** 1 Department of Clinical Chemistry, Erasmus University Medical Center, Rotterdam, Netherlands; 2 Department of Clinical Chemistry, Amsterdam University Medical Center, Amsterdam, Netherlands; 3 Prinses Maxima center for pediatric oncology, Utrecht, Netherlands; 4 Department of Rheumatology, Erasmus University Medical Center, Rotterdam, Netherlands; 5 Department of Pediatric Immunology, University Medical Center Utrecht, Wilhelmina Children’s hospital, Utrecht, Netherlands; 6 Department of Rheumatology, Maasstad hospital, Rotterdam, Netherlands; Soroka University Medical Center, ISRAEL

## Abstract

**Objective:**

The objective was to predict insufficient response to 3 months methotrexate (MTX) in DMARD naïve rheumatoid arthritis patients.

**Methods:**

A Multivariable logistic regression model of rheumatoid arthritis patients starting MTX was developed in a derivation cohort with 285 patients starting MTX in a clinical multicentre, stratified single-blinded trial, performed in seven secondary care clinics and a tertiary care clinic. The model was validated in a validation cohort with 102 patients starting MTX at a tertiary care clinic. Outcome was insufficient response (disease activity score (DAS)28 >3.2) after 3 months of MTX treatment. Clinical characteristics, lifestyle variables, genetic and metabolic biomarkers were determined at baseline in both cohorts. These variables were dichotomized and used to construct a multivariable prediction model with backward logistic regression analysis.

**Results:**

The prediction model for insufficient response in the derivation cohort, included: DAS28>5.1, Health Assessment Questionnaire>0.6, current smoking, BMI>25 kg/m^2^, *ABCB1* rs1045642 genotype, *ABCC3* rs4793665 genotype, and erythrocyte-folate<750 nmol/L. In the derivation cohort, AUC of ROC curve was 0.80 (95%CI: 0.73–0.86), and 0.80 (95%CI: 0.69–0.91) in the validation cohort. Betas of the prediction model were transformed into total risk score (range 0–8). At cutoff of ≥4, probability for insufficient response was 44%. Sensitivity was 71%, specificity 72%, with positive and negative predictive value of 72% and 71%.

**Conclusions:**

A prognostics prediction model for insufficient response to MTX in 2 prospective RA cohorts by combining genetic, metabolic, clinical and lifestyle variables was developed and validated. This model satisfactorily identified RA patients with high risk of insufficient response to MTX.

## Introduction

Methotrexate (MTX) is an anchor-drug in the treatment of rheumatoid arthritis (RA), because of its safety and efficacy.[1, 2] However, in significant numbers of patients, MTX does fail to achieve adequate suppression of disease activity.[3] According to the European league against rheumatism (EULAR) recommendations, therapy should be adjusted in patients who do not reach the treatment target, either remission or low disease activity, after 6 months of therapy;[1, 2] or if no improvement has been achieved within 3 months of MTX treatment.[1] Adaptation of treatment strategy as early as 3 months after MTX start is in line with the need for aggressive and individualised treatment in order to achieve early remission, thus following the clinical practise, in which patients receive early step-up treatment already after 3 months of treatment. Adjustment of therapy in non-responders mostly concerns switching to biologicals, alone or in combination with MTX.[2] Prediction of MTX non-response before MTX start is paramount since first months upon diagnosis represent a window of opportunity during which outcomes can be more effectively modulated by therapy.[4] It is necessary to identify non-responders at baseline in order to ensure that only patients unresponsive to MTX receive early additional treatment with biological or other disease modifying anti-rheumatic drugs (DMARD) and those responsive to MTX are spared feasible costly biologicals.[2]

Prediction models for MTX non-response have been developed earlier for juvenile idiopathic arthritis (JIA)[5] and RA.[6–9] However, these models did not use metabolic predictors and the model developed for RA was not validated and only moderately discriminated responders from non-responders,[6–8] and used the 6-month time-point remission or clinical response as outcome measure. In the RA prediction, there is need for earlier prediction of insufficient response to MTX in order to achieve early remission. Therefore, the aim of this study was to predict insufficient response to 3 months MTX treatment in RA patients before DMARD initiation.

## Methods

### Study design and patients

**Fig 1:**
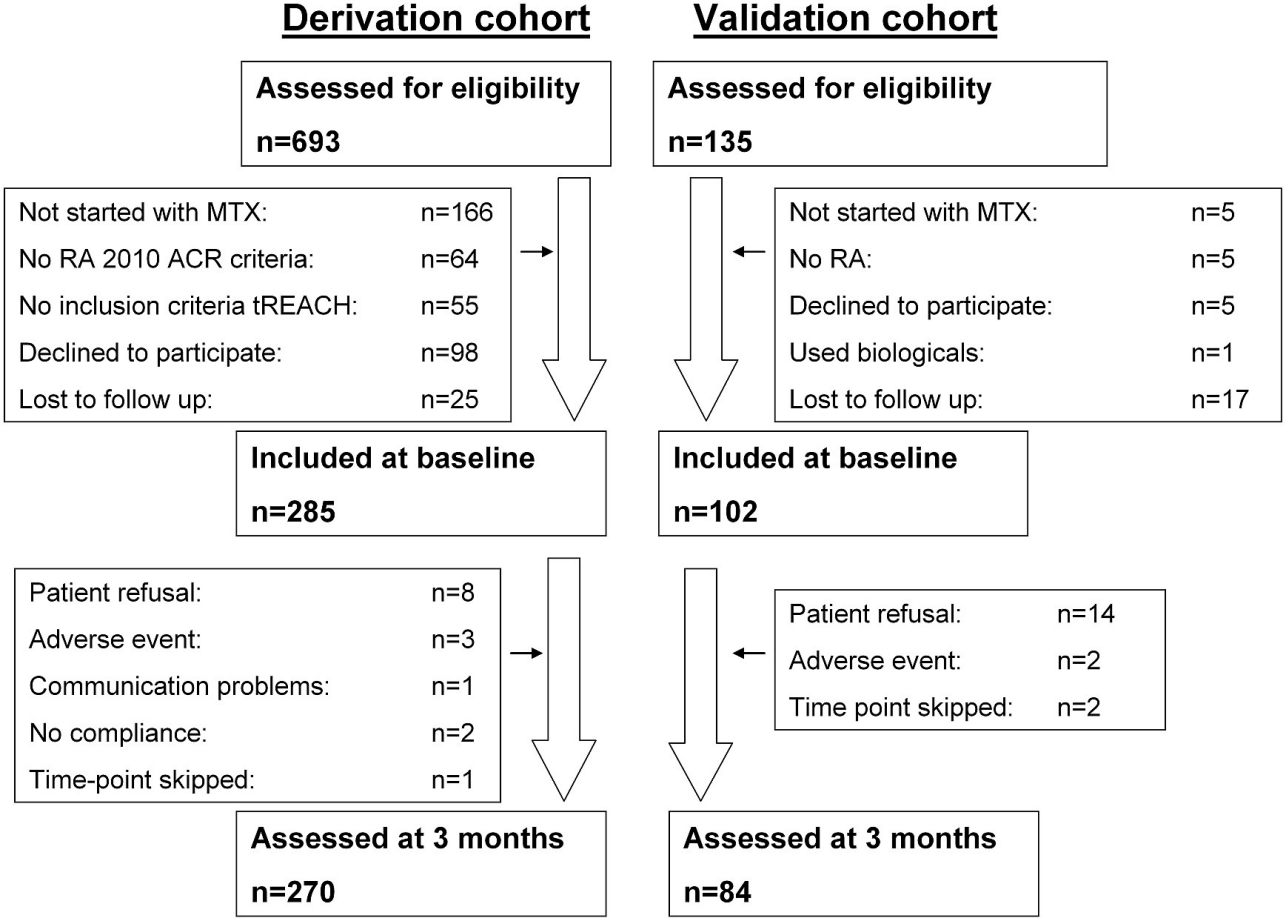
Flow chart of patient follow up divided in the derivation and validation cohorts. MTX, methotrexate; RA, rheumatoid arthritis; ACR, American College of Rheumatology; tREACH, treatment in Rotterdam Early Arthritis Cohort.

This study followed the rules of Transparent Reporting of a multivariable prediction model for Individual Prognosis Or Diagnosis (TRIPOD).[10] Data from two prospective cohorts with Caucasian patients were used. The derivation cohort to construct the prediction model consisted of patients who were enrolled in the treatment in Rotterdam Early Arthritis Cohort (tREACH). This is a clinical multicentre, stratified single-blinded trial (ISRCTN26791028) described elsewhere.[11] 285 DMARD naïve patients starting MTX between July 2007 and October 2011 were selected for this study (Fig 1). The external validation cohort consisted of 102 patients from the Methotrexate in Rotterdam (Netherlands) cohort (MTX-R) who started MTX between January 2006 and December 2010 in the department of Rheumatology, Erasmus University Medical Center, Rotterdam (Erasmus MC), the Netherlands (Fig 1).[12] The medical ethics committee from the Erasmus MC approved both studies and patients gave written informed consent before inclusion.

The derivation cohort included patients from the tREACH who were on MTX and fulfilled the 2010 American College of Rheumatology (ACR) / European League Against Rheumatism (EULAR) criteria for RA. Therefore, only tREACH patients from the high probability group and intermediate probability group A were used since only these patients groups were on MTX.[11] These patients were included in seven secondary care centres and one tertiary care (Erasmus MC) centre in the South-West of the Netherlands.[13] Patients in the validation cohort were included when diagnosed with RA by the physician at the tertiary care centre (Erasmus MC). Inclusion and exclusion criteria for both cohorts are shown in S1 Table. Patients from the derivation cohort started with 25 mg/week MTX and glucocorticoids (GCs) and were randomized to treatment with or without sulfasalazine and hydroxychloroquine. In the derivation cohort, DMARD dosages were: MTX: 25 mg/week orally (dosage reached after 3 weeks), sulfasalazine 2 g/day and hydroxychloroquine 400 mg/day. GCs were either given IM (methylprednisolone 120 mg or triamcinolone 80 mg) or as an oral tapering scheme (week 1–4: 15 mg/day, week 5–6: 10 mg/day, week 7–8: 5 mg/day, and week 9–10: 2.5 mg/day).

In the validation cohort, the physician was free to choose dosing and co-medication. In both cohorts, all patients received folic acid (10 mg/week) during MTX treatment.

### Assessment of insufficient response

Primary outcome was disease activity score 28 (DAS28) at 3 month follow-up, which included 28 tender joint count (TJC), 28 swollen joint count (SJC), visual analog scale (VAS) for general health and the erythrocyte sedimentation rate (ESR).[14] Physicians used the cut-off values of DAS28>3.2 to step-up therapy after 3 months, because low disease activity was not reached. Therefore, insufficient response was defined as DAS28>3.2 after three months of treatment with MTX. DAS28 was assessed by (research) nurses, while being blinded for the study outcome measure.

### Data collection

All potential predictors were associated with MTX inefficacy in previous association studies[5–9, 12, 15, 16] or were likely to be associated based on the physiology (hypothesis-driven approach). All predictors were dichotomized according to commonly used cut-off values or by dividing them into quartiles. A cut-off was chosen at the quartile that had the strongest association with insufficient response as indicated by the highest odds ratio or -2Log likelihood.

### Clinical and lifestyle variables

All predictors were assessed at baseline/diagnosis before initiation of therapy. Before the treatment was started, we collected blood from each RA patient and determined the ESR, C-reactive protein (CRP), TJC, SJC, VAS and DAS28. DAS28>5.1 was used as dichotomous variable for high disease activity at baseline according to the European League Against Rheumatism (EULAR) criteria for response.[17] The Health Assessment Questionnaire (HAQ) was added as variable as it could possibly predict MTX response since mild functional impairment was associated with RA remission.[15]

Lifestyle variables included body mass index (weight (kg)) / (height (m))^2^, smoking, consumption of alcohol and caffeine extracts (Coca-cola, coffee and tea). Caffeine is an adenosine receptor antagonist, which could diminish the anti-inflammatory effect of adenosine, thought to be stimulated by MTX, and therefore possibly decreases MTX response.[18] BMI and smoking were not measured in the validation cohort.

### Metabolic and genetic variables

Metabolic variables were erythrocyte-folate, serum-folate, plasma-homocysteine, erythrocyte-vitamin B_6_, serum-vitamin B_12_, and estimated glomerular filtration rate (eGFR) calculated with the modification of diet in renal disease (MDRD) formula.[19]

Three research blood sample-tubes were obtained during every study visit besides the routine blood samples for erythrocyte sedimentation rate (ESR), C-reactive protein (CRP), Alanine-aminotransferase (ALAT), leukocytes and thrombocytes. One serum tube was centrifuged for 10 min at 1700 g, 4°C; serum was divided into aliquots and stored at -80°C. One EDTA tube was immediately put on ice after collection, centrifuged for 10 min at 1700 g, 4°C, and plasma and cell-pellet aliquots were stored at -80°C. One EDTA tube was kept at room temperature and whole-blood was divided into aliquots and stored at -80°C.

Homocysteine was determined in EDTA-plasma using isotope-dilution liquid chromatography tandem mass spectrometry (LC-MS/MS; waters Acquity UPLC Quattro Premier XE) by an adapted method.[20] For chromatographic separation, a Waters Symmetry C_8_ column 2.1x100mm (Waters, Etten-Leur, Netherlands) with a precolumn (Waters) was used. Vitamin B12 and folate in serum were measured using an electrochemiluminescence immunoassay (Modular E170, Roche, Almere, Netherlands). Vitamin B6 was measured in whole blood with an isotope-dilution LC-MS/MS assay that we described elsewhere.[21] For the erythrocyte-folate assay, 100 μl whole blood was diluted with 1600 μl of a 10 g/l, pH 4, ascorbic acid solution and incubated 3 hours at room temperature. Tubes were centrifuged at 2000 g and analysed with an electrochemiluminescence immunoassay for folate (Modular E170, Roche). Erythrocyte-folate was measured in whole blood from the room temp EDTA tube within 24 hours after sample collection. Erythrocyte-folate stability at room temperature has been proven up to 24 hours.[22] Erythrocyte-folate was corrected for serum-folate and hematocrit. Routine haematology parameters were measured using a Sysmex XE-2100 and ESR was measured using an InteRRliner (Sysmex, Etten-Leur, Netherlands). Routine chemistry parameters were measured on a Roche Modular P analyser (Roche). eGFR was added as possible predictor of MTX outcome, because it influences intracellular MTX polyglutamate concentrations.[23]

Genetic variables consisted of single nucleotide polymorphisms (SNP) were selected based on their involvement in the MTX metabolic pathways, their high polymorphic allele frequency and documented functional effects. In weekly low-dose MTX treatment, MTX polyglutamates accumulate intracellularly and as such inhibit several key enzymes in the folate metabolism and de novo purine synthesis.[16] MTX polyglutamates correlate with MTX efficacy in RA.[16] Non-responders accumulate fewer MTX polyglutamates in red blood cells compared to responders in an early phase of treatment.[16] SNP in genes involved in MTX transport and polyglutamylation affect intracellular MTX accumulation. Inside cells, MTX-PGs inhibit key-enzymes in one-carbon metabolism which is responsible for its therapeutic effects as well as its adverse-event profile. Intracellular MTX prevents cell proliferation and DNA-methylation by displacing the preferred substrates of the folate-dependent enzymes.[24] DNA was obtained from whole blood. SNP selection, DNA isolation and genotyping were performed as we described earlier.[5, 16] All SNPs were determined using real-time PCR with Taqman technique. All laboratory parameters were measured at the clinical chemistry laboratory by technicians blinded for the study.

### Statistical analysis

To construct a model to predict 3 months insufficient response, backward logistic regression analysis was performed in several stages. First, all continuous variables were dichotomized to facilitate the use of the models in daily clinical practice. Second, univariable odds ratios (ORs) with 95% confidence intervals (CI) were calculated. Third, potential predictors (p<0.20) were combined into a multivariable logistic regression model. The full model was simplified according to statistical strength (exclusion if p≥0.200, in each step deleting the variable with the highest p-value), correlations between predictors and practical considerations. If two potential predictors correlated strongly (Spearman’s r≥0.40), the variable that was clinically more relevant or stronger associated with the outcome measure in univariate analysis was given preference.

To calculate predicted probabilities of 3 months DAS28>3.2, we used the following formula:

$$P_{MTXoutcome}=\frac{e^{\left(\beta_{0}+\beta_{1}\cdot x_{1}+\beta_{2}\cdot x_{2}+\ldots +\beta_{p}\cdot x_{p}\right)}}{1+e^{\left(\beta_{0}+\beta_{1}\cdot x_{1}+\beta_{2}\cdot x_{2}+\ldots +\beta_{p}\cdot x_{p}\right)}}$$

were P is the predicted probability of achieving 3 months DAS28>3.2, β_0_ is the constant and β_1_, β_2_ and β_p_ represent the regression coefficients for each of the predictors x_1_, x_2_ and x_p_.

To evaluate the predictive power of the model, we used the predicted probabilities of insufficient response to construct a receiver operating characteristic (ROC) curve. The area under the ROC curve (AUC) measured the concordance of predicted values with actual outcomes, with an AUC of 0.5 reflecting no predictive power and an AUC of 1.0 reflecting perfect prediction. To assess whether the models fit the data well, we used the Hosmer-Lemeshow test.

To compute the risk score of being an insufficient responder to MTX for individual patients, the regression coefficients (β) of the predictors in the final model were transformed into simple scores that sum up to a total risk score. The total risk scores and probabilities of MTX insufficient response for each patient from the derivation cohort was computed. Mean probabilities for each risk score were calculated. Sensitivity, specificity, positive predictive value (PPV) and negative predictive value (NPV) were calculated for each risk score cut-off by using the ROC curve of the derivation cohort. No model updating was performed based on the validation.

The prediction model was externally validated in the validation cohort. The regression coefficients of the predictors obtained from the derivation cohort were entered in the above-mentioned formula. This was used to construct a ROC curve for the validation cohort. All statistical analyses were carried out with SPSS V.21.0.0.1 (SPSS, Chicago, Illinois, USA). No internal validation was performed because an external validation cohort was available. Furthermore, the MTX-R external validation cohort was initiated before the tREACH study and was primarily designed to develop prediction models for MTX efficacy and to investigate pharmacokinetics and dynamics.[12, 25] This study was targeted at a minimum of 100 patients in order to be able to include √100 = 10 predictors in the multivariable prediction model. Hence, there are no accepted approaches to estimate the sample size for prediction model development and validation. The sample size of the derivation cohort was based on the primary objective of the tREACH study.[11, 26] The total number of patients (n = 387) starting MTX and the number of patients that responded insufficient to MTX (n = 148) far exceeded the minimum number needed to develop a multivariable prediction model with 8–10 predictors. Therefore, this study was used to develop the prediction model and the MTX-R study, that was initiated earlier, was used for validation. Because of this, current smoking and BMI were not available in the validation cohort. Because the validation cohort has less parameters than the derivation cohort, model updating / recalibration arising from the validation cohort was not possible.

Missing data was not imputed in the tREACH or MTX-R. If data of a patient were lacking, the patient was left out of the analysis.

## Results

### Patient characteristics

Three months data of the derivation and validation cohorts have been previously published.[12, 26] For the present study, 285 patients from the derivation cohort participated at baseline, of whom 270 also participated at 3 months (Fig 1). From the validation cohort, 102 patients were included at baseline of which 84 participated after 3 months. MTX dose was higher in the derivation cohort as compared to the validation cohort (25 versus 15 mg/week) (Table 1). Patients in the validation cohort had lower DAS28, used more non-steroidal anti-inflammatory drugs (NSAID), less glucocorticoids and received more often MTX as subcutaneous injections than patients in the derivation cohort.[12]

**Table 1:** Baseline characteristics per cohort [12].

Laboratory parameters	Derivation cohort (n = 285)	Validation cohort (n = 102)	p
Plasma-homocysteine (μmol/l), median (IR)	11 (10–14)	12 (10–16)	0.264
Serum-vitamin B12 (pmol/l), median (IR)	290 (231–404)	286 (230–376)	0.588
Serum-folate (nmol/l), median (IR)	17 (13–24)	17 (13–23)	0.742
Erythrocyte-vitamin B6 (nmol/l), median (IR)	80 (64–97)	74 (64–102)	0.485
Erythrocyte-folate (nmol/l), median (IR)	844 (662–1165)	1079 (868–1326)	<0.001
Rheumatoid factor positive	66%	41%	<0.001
Anti-cyclic citrullinated peptide antibody positive	70%	41%	<0.001
Erythrocyte sedimentation rate, mm/h, median (IR)	23 (13–40)	19 (9–33)	0.011
C-reactive protein, mg/l, median (IR)	8 (4–23)	7 (3–14)	0.444
**Clinical parameters**			
Gender, male	30%	29%	0.991
Age, mean (SD)	54 (14)	52 (16)	0.299
VAS mm, mean (SD)	53 (22)	54 (26)	0.704
28 tender joint count, median (IR)	6 (3–10)	4 (1–8)	<0.001
28 swollen joint count, median (IR)	6 (3–10)	3 (1–7)	<0.001
DAS28, mean (SD)	4.94 (1.15)	4.26 (1.43)	<0.001
**Medication**			
Methotrexate dose, mean (SD)	25 (1)	15 (2)	<0.001
NSAIDs	14%	36%	<0.001
Other DMARDs	62%	57%	0.408
Oral corticosteroids	62%	11%	<0.001
Parenteral corticosteroids	32%	3%	<0.001
Subcutaneous methotrexate injections	0%	6%	<0.001

IR, interquartile range; SD, standard deviation; VAS, patient global assessment of general health on a visual analogue scale; DAS, disease activity score; NSAID, non-steroidal anti-inflammatory drug; DMARD, disease modifying anti-rheumatic drug.

In both cohorts, disease activity decreased over time.[12] In the derivation cohort, mean DAS28 was 4.94 (SD = 1.15) at baseline and decreased to 3.12 (SD = 1.19) after three months. In the validation cohort, DAS28 decreased from 4.26 (SD = 1.43) to 2.92 (SD = 1.23). In the derivation cohort (mean DAS28 = 3.12) and validation cohort (mean DAS28 = 2.92), DAS28 was comparable (p = 0.174) after three months of treatment. In the derivation cohort, 116 patients (43%) had a DAS28>3.2 after 3 months and in the validation cohort, 32 patients (38%).

### Prediction model for insufficient response to MTX

**Fig 2:**
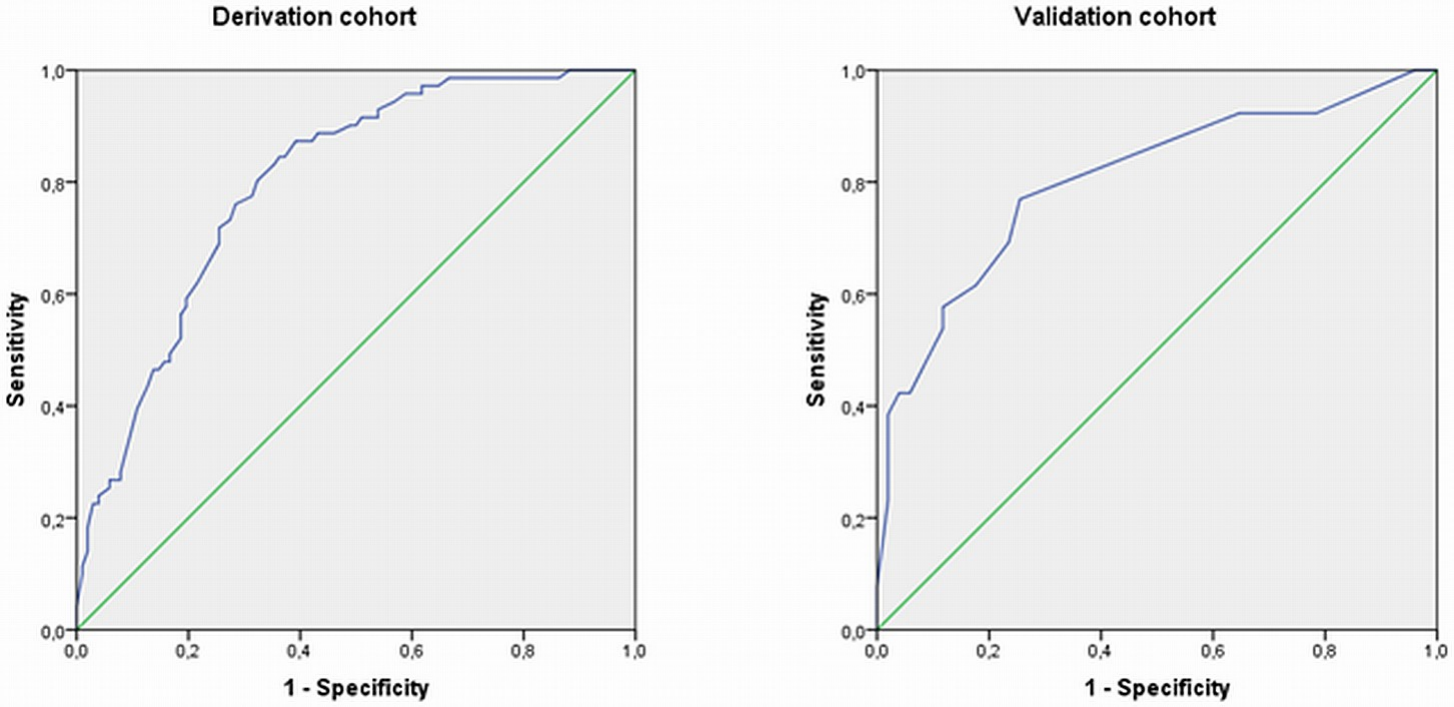
ROC curves for the prediction models of MTX insufficient response (DAS28>3.2) in the derivation (left panel) and validation (right panel) cohorts. Area under receiver operating characteristics curve was 0.80 (95%CI: 0.73–0.86) in the derivation cohort and 0.80 (95%CI: 0.69–0.91) in the validation cohort.

Table 2 shows the SNPs and other baseline variables that were univariabely associated (p≤0.20) with insufficient response after 3 months of therapy in the derivation cohort. These variables were included in the multivariable logistic regression model with backward selection. The variables that remained in the final prediction model were: Adenosine triphosphate Binding Cassette transporter (*ABC*) family B member 1 (*ABCB1)* rs1045642 genotype, *ABCC3* rs4793665 genotype, erythrocyte-folate<750 nmol/L, baseline DAS28>5.1, baseline HAQ>0.6, current smoking and BMI>25 kg/m^2^. The AUC of the prediction model was 0.80 (95% CI: 0.73–0.86), indicating that it classified 80% of patients correctly (Table 3; Fig 2). The Hosmer-Lemeshow goodness-of-fit test was not statistically significant (p = 0.82, indicating that the model fits the data well.

**Table 2:** Prevalencey, univariable OR’s (95% CI) for potential predictors of 3 months DAS28>3.2 insufficient response for derivation and validation cohorts.

Predictors		Derivation cohort		Validation cohort	
		n (%)	OR (95% CI)	n (%)	OR (95% CI)
Female		201 (71)	1.70 (0.99–2.91)*	72 (71)	2.29 (0.80–6.59)*
Age > 40 year		239 (84)	1.76 (0.89–3.50)*	78 (77)	1.44 (0.49–4.28)
**Medication**					
No HCQ		98 (38)	1.47 (0.87–2.48)*	53 (53)	1.05 (0.43–2.54)
No Sulfasalazine		99 (38)	1.42 (0.84–2.40)*	59 (58)	1.51 (0.61–3.77)
No TDT		99 (38)	1.42 (0.84–2.40)*	69 (68)	1.24 (0.47–3.26)
GC		242 (93)	1.68 (0.56–4.98)	14 (14)	2.22 (0.55–8.98)
No IM-GC		160 (62)	1.42 (0.82–2.46)	11 (11)	0.61 (0.04–10.07)
NSAID		37 (14)	1.53 (0.69–3.41)	36 (36)	1.69 (0.67–4.26)
**SNPs (MAF)**					
*ABCB1* rs1045642 G>A (A = 0.3952)	AA vs GG/GA	190 (73)	1.77 (0.99–3.18)*	64 (67)	2.08 (0.71–6.06)*
*ABCB1* rs1128503 G>A (A = 0.4161)	AA vs GG/GA	217 (83)	1.49 (0.76–2.95)	73 (77)	1.21 (0.37–3.93)
*ABCB1* rs2032582 C>A/T (A = 0.3343)	AA/AT/TT vs CC/CA/CT	208 (79)	1.70 (0.90–3.22)*	72 (76)	0.96 (0.31–2.97)
*ABCC1* rs35592 T>C (C = 0.3704)	TC/CC vs TT	163 (62)	1.05 (0.63–1.75)	55 (58)	0.95 (0.37–2.43)
*ABCC1* rs3784862 A>G (G = 0.4155)	AG/GG vs AA	153 (58)	1.05 (0.63–1.75)	43 (45)	0.52 (0.20–1.36)*
*ABCC2* rs717620 C>T (T = 0.1350)	CC vs CT/TT	74 (28)	1.30 (0.75–2.25)	30 (32)	0.77 (0.28–2.11)
*ABCC2* rs4148396 C>T (T = 0.3147)	CC/CT vs TT	35 (13)	1.46 (0.71–3.01)	10 (11)	1.47 (0.30–7.10)
*ABCC3* rs3785911 A>C (C = 0.2452)	AC/CC vs AA	134 (51)	1.28 (0.78–2.11)	45 (47)	1.41 (0.55–3.59)
*ABCC3* rs4793665 T>C (C = 0.3313)	TT vs TC/CC	173 (66)	2.02 (1.17–3.49)*	68 (72)	0.36 (0.13–1.00)*
*ABCC4* rs868853 T>C (C = 0.1757)	TT vs TC/CC	38 (14)	1.21 (0.60–2.45)	20 (21)	1.53 (0.53–4.43)
*ABCC4* rs2274407 C>A (A = 0.1506)	CA/AA vs CC	232 (88)	1.09 (0.50–2.38)	81 (85)	0.59 (0.16–2.14)
*ABCC5* rs2139560 G>A (A = 0.3027)	AA vs GG/GA	212 (81)	1.52 (0.79–2.90)	78 (82)	1.23 (0.34–4.44)
*ABCG2* rs2231142 G>T (T = 0.1194)	GT/TT vs GG	204 (78)	1.54 (0.83–2.87)*	78 (82)	7.15 (0.87–58.73)*
*ABCG2* rs13120400 T>C (C = 0.1052)	TT vs TC/CC	110 (42)	1.08 (0.65–1.79)	37 (39)	1.56 (0.61–4.02)
*ADA* rs73598374 C>T (T = 0.0513)	CC vs CT/TT	33 (13)	1.68 (0.80–3.54)*	13 (14)	1.22 (0.36–4.18)
*ADORA2A* rs5751876 C>T (C = 0.4423)	TT vs CC/CT	213 (81)	1.17 (0.62–2.22)	81 (85)	0.18 (0.04–0.76)*
*AMPD1* rs17602729 G>A (A = 0.0381)	GG vs GA/AA	45 (17)	1.49 (0.78–2.86)	17 (18)	0.46 (0.12–1.80)
*ATIC* rs2372536 C>G (G = 0.2778)	GG vs CC/CG	226 (86)	1.69 (0.79–3.61)*	86 (91)	4.14 (0.48–35.53)*
*FOLR2* rs514933 T>C (C = 0.4285)	TT/TC vs CC	42 (16)	1.24 (0.63–2.43)	16 (17)	1.06 (0.32–3.55)
*FPGS* rs4451422 A>C (C = 0.4643)	AA/AC vs CC	61 (23)	1.46 (0.80–2.66)	19 (20)	1.73 (0.59–5.07)
*GGH* rs3758149 G>A (A = 0.2316)	GG vs GA/AA	137 (52)	1.58 (0.95–2.61)*	41 (43)	0.90 (0.35–2.30)
*GGH* rs10106587 A>C (C = 0.2576)	AC/CC vs AA	134 (51)	1.02 (0.62–1.69)	43 (45)	0.77 (0.30–1.99)
*ITPA* rs1127354 G>T (A = 0.0895)	GT/TT vs GG	229 (87)	1.04 (0.50–2.16)	79 (83)	1.07 (0.29–3.94)
*MTHFR* rs1801131 A>C (G = 0.2494)	AA vs AC/CC	139 (53)	1.37 (0.83–2.27)	42 (44)	0.97 (0.38–2.49)
*MTHFR* rs1801133 C>T (A = 0.2454)	CT/TT vs CC	125 (48)	1.61 (0.97–2.66)*	55 (58)	0.68 (0.26–1.77)
*MTRR* rs1801394 A>G (G = 0.3642)	GG vs AA/AG	174 (66)	1.45 (0.85–2.47)*	67 (71)	0.52 (0.19–1.44)
*SLC19A1* rs1051266 C>T (C = 0.4886)	TT vs CC/CT	220 (84)	1.48 (0.73–2.98)	77 (81)	2.20 (0.56–8.69)
*SLC46A1* rs2239907 C>T (T = 0.4515)	CC/CT vs TT	52 (20)	1.94 (1.04–3.60)*	13 (14)	1.30 (0.33–5.09)
**Metabolic parameters**					
Erythrocyte-folate<750 nmol/L		74 (37)	1.48 (0.82–2.69)*	14 (14)	2.80 (0.80–9.79)*
Serum-folate<13 nmol/L		57 (24)	1.43 (0.77–2.66)	25 (25)	0.95 (0.33–2.74)
Plasma homocysteine>14 μmol/L		64 (27)	1.16 (0.64–2.11)	34 (34)	1.10 (0.43–2.81)
Serum vitamin B_6_<80 nmol/L		103 (50)	1.40 (0.80–2.47)	57 (58)	1.23 (0.50–3.03)
Serum vitamin B_12_>400 pmol/L		61 (26)	1.11 (0.61–2.03)	20 (20)	2.30 (0.78–6.82)*
eGFR<80 ml/min/1.73m^2^		63 (53)	1.43 (0.67–3.03)	39 (38)	1.35 (0.55–3.32)
**Disease activity**					
ESR>40 mm/hour		73 (26)	2.77 (1.58–4.85)*	18 (18)	1.83 (0.61–5.50)
CRP>10 mg/L		125 (44)	1.37 (0.74–2.54)	31 (30)	0.81 (0.31–2.13)
TJC>3 joints		221 (80)	3.81 (1.95–7.43)*	58 (57)	3.78 (1.44–9.97)
SJC>3 joints		229 (80)	2.05 (1.08–3.89)*	55 (54)	1.50 (0.62–3.64)
VAS>34 mm		215 (75)	3.82 (2.00–7.32)*	77 (76)	1.44 (0.49–4.58)
DAS28>5.1		125 (44)	3.70 (2.23–6.16)*	25 (25)	5.96 (1.98–17.93)*
HAQ>0.6		217 (76)	2.80 (1.51–5.18)*	88 (86)	1.27 (0.35–4.63)
Rheumatoid factor negative		85 (34)	1.58 (0.92–2.72)*	55 (59)	0.96 (0.38–2.47)
Anti-CCP negative		75 (30)	1.41 (0.81–2.46)	55 (59)	1.68 (0.65–4.37)
Disease duration>145 days		143 (51)	1.10 (0.68–1.78)	**	**
**Life style**					
Smoking		87 (33)	2.01 (1.19–3.41)*	**	**
Alcohol consumption<30 glasses/month		191 (73)	1.47 (0.84–2.58)*	34 (76)	2.25 (0.39–13.17)
Cola consumption>30 glasses/month		33 (13)	1.28 (0.62–1.67)	16 (16)	1.64 (0.53–5.08)
Coffee consumption<90 glasses/month		117 (45)	1.11 (0.68–1.83)	50 (51)	1.19 (0.48–2.93)
Tea consumption>90 glasses/month		57 (22)	1.58 (0.87–2.88)*	20 (20)	0.19 (0.04–0.90)*
BMI>25 kg/m^2^		157 (56)	1.70 (1.04–2.79)*	**	**

*Variables significantly associated with MTX non-response (p<0.200) in the derivation cohort were included in the multivariate backward logistic regression analysis;

**not determined;

HCQ, hydroxychloroquine; TDT, triple disease-modifying antirheumatic drug therapy; GC, glucocorticoids; IM, intramuscular; NSAID, non-steroidal anti-inflammatory drugs; *ABCB1*, Adenosine triphosphate-binding cassette transporter B1; *ADA*, adenosine-deaminase; *ADORA2A*, adenosine A2A receptor; *AMPD1*, adenosine monophosphate deaminase 1; *ATIC*, 5-aminoimidazole-4-carboxamide ribonucleotide transformylase; *FOLR2*, Folate receptor 2; *FPGS*, folylpolyglutamate synthetase; *GGH*, γ-glutamyl hydrolase; *ITPA*, inosine triphosphatase; *MTHFR*, methylenetetrahydrofolate reductase; *MTRR*, methionine synthase reductase; *SLC19A1*, solute carrier 19A1; eGFR, estimated glomerular filtration rate; TJC, tender joint score in 28 joints; SJC, swollen joint score in 28 joints; HAQ, health assement questionnaire; CCP, cyclic citrulinated peptide; BMI, body mass index; MAF, Global minor allele frequency in NCBI SNP database.

**Table 3:** Prediction model and scores for 3 months MTX non-response (DAS28>3.2).

Predictors		β	Score	OR (95% CI)	p
Baseline Das28	>5.1	1.13	1	3.08 (1.26–7.52)	0.014
HAQ	>0.6	1.26	1	3.53 (1.30–9.56)	0.013
*ABCB1* rs1045642 G>A	AA vs GG/GA	1.34	1	3.82 (1.54–9.47)	0.004
*ABCC3* rs4793665 T>C	TT vs TC/CC	1.24	1	3.45 (1.44–8.31)	0.006
Folate in erythrocytes	<750 nmol/L	0.82	1	2.28 (1.02–5.12)	0.046
Current Smoking		1.75	2	5.77 (2.34–14.24)	<0.001
BMI	>25 kg/m^2^	1.11	1	3.02 (1.31–6.97)	0.009
Constant		-5.07	*	0.01	
AUC derivation cohort				0.80 (0.73–0.86)	<0.001
AUC validation cohort				0.80 (0.69–0.91)	<0.001
Hosmer-Lemeshow test				0.816	

Risk score of an RA patient having all predictors is calculated as follows: Add up scores of individual predictors, namely 1+1+1+1+1+2+1, which equals 8 points.

*The constant was suppressed.

MTX, methotrexate; DAS28, disease activity score in 28 joints; OR, odds ratio; CI, confidence interval; HAQ, health assessment questionnaire; *ABCB1*, adenosine triphosphate-binding cassette transporter B1; BMI, body mass index; AUC, area under the receiver operating characteristics curve.

These predictors were used to test the model in the validation cohort. Smoking and BMI were not determined in the validation cohort, and therefore could not be tested. The AUC in the validation cohort also was 0.80 (95% CI: 0.69–0.91) (Table 3) indicating that 80% of patients were classified correctly. The AUC of 0.80 of the validation cohort fits within the 95% CI of the AUC of the developmental cohort (0.73–0.86), indicating that the AUC’s of both cohorts are not statistically different.

To make our prediction model suitable for daily practice, we transformed the regression coefficients (β) of the model’s predictors, into simple scores. Thereafter, individual risk scores for having DAS28>3.2 after 3 months of therapy were computed (Table 4). The constant (beta = -5.07) of the multivariable model of -5 was suppressed in order to simplify the model and not used. The score ranged from 0 to 8 whereby a higher score reflected higher chance at treatment failure (insufficient response) after 3 months. Risk score of a patient, who had all predictors of the final model, was calculated by adding up simple scores, assigned to individual predictors: 1+1+1+1+1+2+1, which results in a risk score of 8. If all predictors were present the probability of 3 months DAS28>3.2 is 0.80. The risk score of a patient having no predictors would be equal to 0. If no predictors were present, the probability of insufficient response was 0.01. Within the 0–8 range, the diagnostic accuracy of different cut-offs for the prediction model was evaluated by calculating the risk scores, and probability of having insufficient response, for each individual patient in the derivation cohort.

**Table 4:** Diagnostic parameters for various risk score cut-offs predicting MTX insufficient response (3 months DAS28>3.2) in the derivation cohort.

Cut-off	Probability (%)*	Sensitivity (%)	Specificity (%)	PPV (%)	NPV (%)
≥1	10	99	27	58	96
≥2	20	97	38	61	93
≥3	33	86	63	70	82
≥4	44	71	72	72	71
≥5	58	47	85	76	62
≥6	56	47	85	76	62
≥7	85	11	99	92	53
≥8	80	14	98	88	53

Risk scores were calculated in each patient in the derivation cohort.

*mean probability of all patients with the specific risk score for insufficient response (3 months DAS28>3.2).

MTX, methotrexate; DAS, disease activity score; PPV, positive predictive value; NPV, negative predictive value.

When only DAS28>5.1 was included the AUC under the ROC curve was 0.66 (95% CI: 0.59–0.72) in the derivation cohort, and 0.66 (95% CI: 0.54–0.79) in the validation cohort. When HAQ>0.6, erythrocyte folate<750 nmol/L, *ABCB1 rs1045642* and *ABCC3* rs4793665 were added, the AUC raised to 0.73 (95% CI: 0.66–0.80) in the derivation and to 0.80 (95% CI: 0.69–0.91) in the validation cohort. When, finally, current smoking and BMI>25 were added to the prediction model in the derivation cohort, the AUC rose to 0.80 (95% CI: 0.73–0.86).

## Discussion

We developed and validated a model, which could predict insufficient response to MTX after 3 months of therapy, before start of MTX treatment in 2 large prospective cohorts including patients with RA. The model included DAS28>5.1 before start of MTX, HAQ>0.6, *ABCB1* rs1045642 genotype, *ABCC3* rs4793665 genotype, erythrocyte-folate<750 nmol/L, current smoking and BMI>25 kg/m^2^. The model classified 80% of patients correctly in the derivation and validation cohort.

Earlier studies did not include metabolic predictors[6–8] and one lacked a validation cohort.^6^ Our study is the first validated and prospective study on prediction of MTX insufficient response that also incorporated metabolic predictors. One of the earlier reported models to predict MTX efficacy in MTX monotherapy in RA classified 85% of patients correctly.[6] This model was not yet validated. In agreement with our results, this model contained DAS at diagnosis and smoking status and genetic variables. However, in contrast to our study, this study did not assess BMI and folate status as possible predictors. Furthermore, they investigated other genetic factors, namely *AMPD1*, *ATIC*, *ITPA* and *MTHFD1* genotypes, which were not included in our study.

Others[7] developed a prediction model using the EULAR response criteria[17] as dependent variable and current smoking, female gender, longer symptom duration and younger age as independent variables. EULAR response criteria can only be used in patients with baseline DAS28≥3.3 and not in all patients with RA. We also found that women had a higher risk of insufficient response, but this variable was not included in the final model since other variables were stronger predictors.[17] Conflicting results on the association of BMI and disease activity in RA have been reported; underweight and obesity both have been associated with worse disease activity in RA.[27, 28]

The cut-off could be ≥4 (Fig 2, Table 4) with a probability for having 3 months DAS28>3.2 of 0.44. At this cut-off, 71% of the insufficient -responders could have received other therapy (i.e. biologicals) earlier, which may prevent irreversible joint destruction. However, 28% of responders would receive other medication while MTX would have worked. Whether the cost reduction of less irreversible joint destruction exceeds the extra costs that will be made for biologicals should be further investigated. The interest of the rheumatology community is focused more towards biological therapies, although no clinical prediction rules have been published yet to individualize DMARD therapy. Both the ACR[29] and the EULAR[1] recommend MTX as first-line therapy in RA. Methotrexate is first-line therapy and should be prescribed at an optimal dose of 25 mg weekly and in combination with glucocorticoids; 40% to 50% of patients reach remission or at least low disease activity with this regimen.[30] If this treatment fails, sequential application of targeted therapies, such as biologic agents (eg, tumor necrosis factor inhibitors) or Janus kinase inhibitors in combination with methotrexate, have allowed up to 75% of these patients to reach the treatment target over time.[30] As methotrexate is today still the first-line therapy and is much cheaper than biologic agents, the insufficient response will help in making the choice between biologic agents and methotrexate at forehand and minimize losing precious time with bone damage or severe adverse events. Therefore, both prediction rules for first-line MTX non-response and second-line biological response are needed to individualize therapy in RA.

The AUC of the ROC curve of the final model without current smoking and BMI>25 was lower in the derivation cohort (AUC = 0.73 (95% CI: 0.66–0.80)) as compared to the validation cohort (AUC = 0.80 (95% CI: 0.69–0.91) cohort. However, both AUCs are not significantly different since, 0.73 is within the 95% CI of the AUC of the validation cohort. At 6 months 64 of the 178 patients in the derivation and 24 of the 64 patients in the validation cohort had a DAS28 > 3.2. When the model was applied at 6 months after start of MTX, the AUC in the validation cohort was 0.73 (95% CI: 0.65–0.81) and in the validation cohort the AUC was 0.61 (0.46–0.77). Also, at 6 months the AUCs between the two cohorts are not significantly different since 0.73 is within the 95% CI of the AUC of the validation cohort.

A pitfall of this study was that current smoking and BMI>25 could not be replicated in the validation cohort. These variables have to be replicated in other cohorts. However, we decided to keep these predictors in the prediction model, because they are easy assessable and strong predictors improving the predictive value of the model (AUC from 0.73 to 0.80). The impracticality regarding SNP and laboratory predictors could make future clinical utilization difficult. This may turn the current practice of methotrexate trial easier to be done in most of the clinics. The biochemical parameters add complexity to the clinical implementation of the prediction model. However, the biochemical parameters added significant predictive power to the use of clinical parameters alone. Although erythrocyte folate is not available in every laboratory, it is relatively easy to measure using a routine immunochemistry platform. Similarly, SNPs can be measured relatively easily these days using automated DNA extraction equipment and SNP platforms. With current smoking and BMI>25, in addition to DAS28>5.1 and HAQ>0.6, without laboratory predictors, the AUC was 0.76 (95% CI: 0.70–0.82) in the derivation cohort, indicating good predictive value (76% will be classified correctly). Inclusion of the laboratory predictors, erythrocyte-folate<750 nmol/L, *ABCB1 rs1045642* and *ABCC3* rs4793665, improved the AUC to 0.80, indicating that 80% of the patients will be classified correctly. We aimed for the highest possible AUC (AUC = 0.80 (95% CI: 0.73–0.86)) in the derivation cohort and therefore choose to keep the laboratory parameters in the model. Nowadays, it is easy and not expensive to measure SNPs and erythrocyte-folate. In addition, erythrocyte-folate is a routine laboratory test and genotyping might be transformed to fast-test if useful.

Although, 25 mg/week is quite a high dosage that not always is reached world wide, in the derivation cohort mean doses of 25 mg/week (SD: 1 mg/week) were used and 15 mg/week (SD: 2 mg/week) in the validation cohort. Therefore, the prediction rule is generalizable to mean doses of MTX of 15–25 mg/week. The EULAR guideline actually states that MTX should be used in sufficient doses of 25–30 mg/week. Similarly, Methotrexate is first-line therapy and should be prescribed at an optimal dose of 25 mg weekly and in combination with glucocorticoids; 40% to 50% of patients reach remission or at least low disease activity with this regimen.[30] As this is the optimal dose according to current guidelines this should be the dose for which the model must predict the insufficient response.

MTX-dose could not be added as predictor itself because there was no variation in MTX-dose within the cohorts. We showed before that erythrocyte MTX polyglutamate levels are associated with treatment response in JIA[31] and RA.[25] It is worthwhile to examine this a potential predictor in new studies.

A strong point of this study was that the developed prediction model was externally validated and that the predictive value was equally high in the validation cohort. This prediction model should be validated in a trial were insufficient response is predicted before treatment is started and patients treated according to the predicted insufficient response.

In conclusion we developed and validated a prediction model for insufficient response to MTX in 2 prospective RA cohorts by combining genetic, metabolic, clinical and lifestyle variables. This model can satisfactorily identify RA patients with a high risk of insufficient response to MTX after three months of treatment, and can, therefore, be used by clinicians as a tool for personalized treatment. RA patients who are likely to be unresponsive to MTX therapy, may be (additionally) treated with biologicals or other DMARDs without further delay.

## Supporting information

S1 Dataset Anonymized database de rotte et al 20181122.sav. (SAV)

S1 Table Exclusion criteria for the derivation and validation cohort. (DOCX)
