# Supplementary material for: Development and validation of a prognostic multivariable model to predict insufficient clinical response to methotrexate in rheumatoid arthritis
Source: PLoS One. 2018 Dec 10;13(12):e0208534. doi: 10.1371/journal.pone.0208534 (PMC6287811; doi:10.1371/journal.pone.0208534)
Supplement: S1 Table — (DOCX) [file pone.0208534.s002.docx]

**S1 table** Exclusion criteria for the derivation and validation cohort.

| Cohort | Inclusion criteria | Exclusion criteria |
| --- | --- | --- |
| Derivation | Arthritis in ≥ 1 joint  Duration of complaints < 12 months  Age ≥ 18 years  Informed consent  2010 ACR/EULAR criteria for RA | No possibility to communicate  Trauma  Gout, infectious arthritis or a systematic disease  Former anti-rheumatic therapy  Contra indication for study medication |
| Validation | Age ≥ 18 years  Informed consent  Diagnosed with RA by physician  MTX prescribed by physician | No possibility to communicate  Trauma  Gout, infectious arthritis or a systematic disease  Former anti-rheumatic therapy |

ACR, American College for Rheumatology; EULAR, European League Against Rheumatism; RA, rheumatoid arthritis; MTX, methotrexate.
